# Detection of inhibitors of *Candida albicans* Cdr transporters using a diS-C_3_(3) fluorescence

**DOI:** 10.3389/fmicb.2015.00176

**Published:** 2015-03-09

**Authors:** Joanna Szczepaniak, Marcin Łukaszewicz, Anna Krasowska

**Affiliations:** Faculty of Biotechnology, University of Wroclaw, Wroclaw, Poland

**Keywords:** *Candida albicans*, ABC transporters, inhibitors, diS-C_3_(3), enniatin A, beauvericin

## Abstract

*Candida albicans* is a major cause of opportunistic and life-threatening, systemic fungal infections. Hence new antifungal agents, as well as new methods to treat fungal infections, are still needed. The application of inhibitors of drug-efflux pumps may increase the susceptibility of *C. albicans* to drugs. We developed a new fluorescence method that allows the *in vivo* activity evaluation of compounds inhibiting of *C. albicans* transporters. We show that the potentiometric dye 3,3′-dipropylthiacarbocyanine iodide diS-C_3_(3) is pumped out by both Cdr1 and Cdr2 transporters. The fluorescence labeling with diS-C_3_(3) enables a real-time observation of the activity of *C. albicans* Cdr1 and Cdr2 transporters. We demonstrate that enniatin A and beauvericin show different specificities toward these transporters. Enniatin A inhibits diS-C_3_(3) efflux by Cdr1 while beauvericin inhibits both Cdr1p and Cdr2p.

## INTRODUCTION

The mechanism of resistance in yeasts to antifungal drugs is different depending on the mode of action of the antifungals (Spampinato and Leonardi, 2013). The drug-efflux system represented by plasma membrane transporters is one of four mechanisms of multidrug resistance in *Candida albicans* (Sanglard et al., 2009).

Three efflux pumps situated in the *C. albicans* plasma membrane are responsible for decreasing the intracellular concentration of antifungals. These pumps are encoded by *Candida* drug resistance (*CDR1* and *CDR2*) and multidrug resistance (*MDR1*) genes and they differ in the source of energy used for their activity and in the specificity to the antifungals molecules (Cannon et al., 2009).

One of the strategies used to identify the mechanism and function of the *C. albicans* efflux pumps, and to screen for their substrates and inhibitors, is the preparation of the collection of *C. albicans* mutants with deletions of the *CDR1*, *CDR2*, and *MDR1* genes, which are used for investigating the molecular mechanisms governing the regulation of multidrug transporter genes (Coste et al., 2004, 2009). Studies of the multidrug resistance process have provided important knowledge about efflux pump gene regulation, their substrates and inhibitors, sources of energy, and transport mechanism.

Another strategy for testing drugs’ inhibition of the efflux pumps is to study their heterologous expression in the non-pathogenic yeast *Saccharomyces cerevisiae* (Cannon et al., 2009). Tanabe et al. (2011) cloned 28 chimeric constructs between *C. albicans* Cdr1p (CaCdr1p) and Cdr2p (CaCdr2p) into *S. cerevisiae*, showing that most of the transmembrane spans and the nuclear binding domains (NBDs) are inhibitor binding sites or affect substrate efflux. Although *S. cerevisiae* is a frequently chosen yeast organism for expression and investigation of *C. albicans* efflux pumps, it is important to consider the differences in the metabolism of the two microorganisms (Rodaki et al., 2009; Calahorra et al., 2012). Besides the obvious differences between the two species, heterologous expression affects several other intracellular interactions responsible for resistance to drugs. For example, tested transporter expression level, and its interplay with other proteins and regulations systems, could be completely different.

Thus, it is important to develop methods that may enable real-time observation of transporter activity fluctuations in response to environmental factors in wild, not modified strains. To this day the most popular method to measure activity of transporters is using rhodamine 6G or rhodamine 123 (Clark et al., 1996) or nile red as pump subtrates (Ivnitski-Steele et al., 2010). But methods and knowledge about the activity of the pumps in real time is scarce. Therefore, our purpose was to develop such a method and to validate it by using collections of isogenic strains with deletions of *CDR1*, *CDR2*, and *MRD1* genes and by testing transporters inhibitors.

One of the most potent inhibitors of MDR transporters are group of enniatins, cyclic hexadepsipeptides produced by *Fusarium spp.* Those mycotoxins have ionophoric properties but it was shown that enniatin can interact with *S. cerevisiae* Pdr5p (Hiraga et al., 2005) and *C. albicans* Cdr1p (Holmes et al., 2008) and inhabit their activity. Other compound from this family, beauvericin was observed to act synergistically with miconazole (Fukuda et al., 2004) and ketoconazole (Zhang et al., 2007) also suggesting its involvement in ATP binding cassette (ABC) transporters inhibition.

Hendrych et al. (2009) developed a novel screening method which uses potentiometric fluorescent probe diS-C_3_(3) that measures the kinetics and potency of inhibitors of the *S. cerevisiae* multidrug resistance pumps. In this work, we show for the first time in *C. albicans* that diS-C_3_(3) is pumped out of the cell by both Cdr1p and Cdr2p. We set up the method for testing new drugs and transporters inhibitors, and we also demonstrated that enniatin A and beauvericin are effective inhibitors of Cdr1p and both Cdr1p and Cdr2p, respectively.

## MATERIALS AND METHODS

### STRAINS AND GROWTH MEDIA

The *C. albicans* strains used in this study (Table 1) were generous gifts from D. Sanglard (Lausanne, Switzerland; Sanglard et al., 1995, 1997; Sanglard and Ischer, 1996). All strains were grown at 28°C on YPD medium with 2% glucose, 1% Bacto peptone (Difco), and 1% yeast extract (Difco) and they were shaken at 120 rpm, as described herein. Solid medium was supplemented with 1.5% agar.

**TABLE 1:** **Collection of *C. albicans* strains used in this study**.

**Strain**	**Genotype**	**Reference**
CAF 2-1	*ura3Δ::imm434/URA3*	Fonzi and Irwin (1993)
DSY 448	*cdr1Δ::hisG-URA3-hisG/cdr1Δ::hisG*	Sanglard and Ischer (1996)
DSY 465	*mdr1Δ::hisG-URA3-hisG/mdr1Δ::hisG*	Sanglard and Ischer (1996)
DSY 653	*cdr2Δ::hisG-URA3-hisG/cdr2Δ::hisG*	Sanglard et al. (1997)
DSY 654	*cdr1Δ::hisG/cdr1Δ::hisG cdr2Δ::hisG-URA3-hisG/cdr2Δ::hisG*	Sanglard et al. (1997)
DSY 1050	*cdr1Δ::hisG/cdr1Δ::hisG cdr2Δ::hisG/cdr2Δ::hisG mdr1Δ::hisG-URA3-hisG/mdr1Δ::hisG*	Mukherjee and Chandra (2003)

### SAMPLE PREPARATION

Cells were prepared according to Gásková et al. (1998) with modifications. Stationary cultures were prepared by growing strains at 28°C for 24 h. A volume of 150 μl of stationary culture was added to 20 ml of fresh YPD medium, incubated for 10 h at 28°C, and was shaken at 120 rpm. The cells were harvested by centrifuging at 110 × *g* for 3 min, washed twice with deionized water, resuspended in citrate-phosphate (CP) buffer (pH 6.0) at OD_600_ = 0.1 or OD_600_ = 0.4 (±10%), and kept on ice.

### DiS-C_3_(3) UPTAKE INTO CELLS

Aliquots of cell suspensions in CP buffer (3 ml, OD_600_ = 0.1; 1.02 × 10^6^ cfu) were labeled with diS-C_3_(3) (Sigma) at a final concentration of 5 × 10^–8^ M at room temperature. Fluorescence spectra were measured every 4 min for 120 min, with gentle stirring before each measurement, with a Fluorescence Spectrophotometer (HITACHI F-4500) equipped with a xenon lamp. The excitation wavelength was 531 nm and the fluorescence range was 560–590 nm. Scattered light was eliminated by an amber glass filter with a cutoff wavelength of 540 nm. Where indicated herein, 2% glucose was added after 60 min and enniatin A (2 μg/ml) (Sigma) and beauvericin (2 and 0.1 μg/ml) (Cayman) was added after 80 min. All experiments were repeated at least three times and means with standard deviation were used as staining curve.

### DISK DIFFUSION ASSAY

*Candida* cells were suspended in deionized water (McFarland standard No. 0.5) and were streaked on YPG agar plates. Tested antifungal agents at concentrations described herein were applied to sterile OXOID Antimicrobial Susceptibility Test Disks, which were then placed on the agar. Culture growth was assessed after a 48 h incubation at 28°C. In disk diffusion assays concentrations below the one that gave inhibitory effect for a given compound was used 1/2 MIC (minimal inhibitory concentration)for fluconazole determined independently for each strain.

### CONFOCAL MICROSCOPY

Cell suspensions in CP buffer (5 ml, OD_600_ = 0.4) were stained with 2 × 10^–7^ M diS-C_3_(3) probe for 30 and 150 min, with 2% glucose added after 60 min and enniatin A (2 μg/ml) and beauvericin (2–40 μg/ml) added after 80 min. Aliquots of cell suspensions were pelleted by centrifuging, washed in deionized water, and 4 μl of samples were viewed with Leica TCS SP8 X confocal microscope.

## RESULTS

### FLUORESCENT PROBE diS-C_3_(3) IS A SUBSTRATE FOR *C. albicans* Cdr1 AND Cdr2 TRANSPORTERS

**FIGURE 1:**
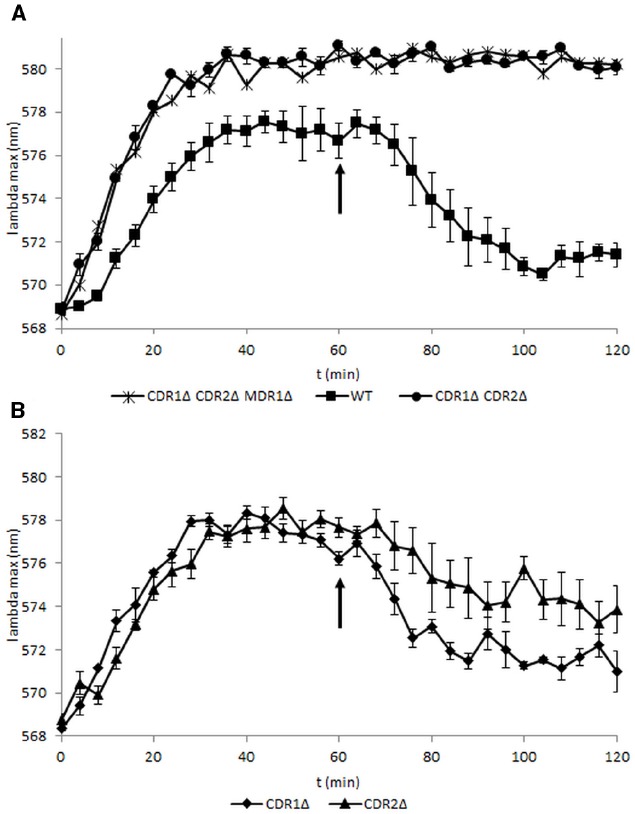
**Fluorescence of diS-C_3_(3) probe in strains with deleted transporters and wild-type strain.** Probe was added at the beginning of the experiment and 2% glucose was added at the 60 min point. Fluorescence in the strains was measured every 4 min. The probe is exported by the ABC transporters Cdr2 and, after addition of glucose, Cdr1—but not by the MFS family transporter Mdr1 (*N* = 3–4).

Previous studies of *S. cerevisiae* have shown that the fluorescent probe diS-C_3_(3) is a substrate for the pleiotropic drug resistance (PDR) pumps, namely the Pdr5p and Snq2p pumps (Hendrych et al., 2009). The observed fluorescence results from both passive membrane-potential-dependent probe uptake and active probe extrusion by ABC pumps. The final maximum fluorescence wavelength (λmax) corresponds to the concentration equilibrium of the probe. Since the λmax of free probe in solution is about 10 nm lower than that of probe bound inside the cell, higher concentration of the probe within the cell results in higher λmax (red shift). The magnitude of this red shift decreases when the probe accumulation in the cell is lowered by the action of probe-expelling pumps; the extent of this lowering thus reflects relative activity of the transporters. To determine whether diS-C_3_(3) is suitable for measuring transporter activity in *C. albicans* and to determine which pumps are responsible for its export, we monitored fluorescence in a collection of strains which expressed all pumps (wild type, WT), or which lacked the Cdr1, Cdr2, or Mdr1 pump (Table 1; Figures 1A,B). A maximum red shift was measured in a strain with simultaneous deletion of both Cdr1p and Cdr2p and a strain lacking all three transporters–Cdr1, Cdr2, and Mdr1. Mutants lacking Cdr1p or Cdr2p stained more intensely than the parent strain (Figure 1A). This means that diS-C_3_(3) is actively expelled from *C. albicans* cells and serves as the substrate for the two Cdr transporters but not for Mdr1.

In the strain lacking Cdr2p the final λmax was higher, and thus the intracellular concentration of the probe was higher than in strain lacking Cdr1p. This indicates that under these conditions Cdr2p plays a larger role in lowering of the probe concentration in stained cells than Cdr1p.

### ENNIATIN A AND BEAUVERICIN INHIBIT TRANSPORTERS WITH DIFFERENT SPECIFICITY

**FIGURE 2:**
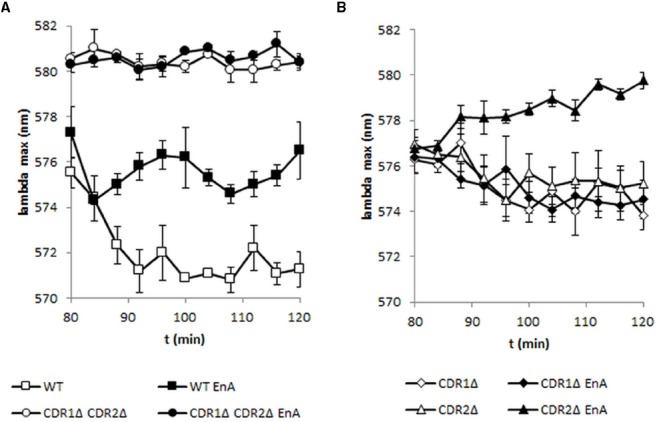
**Inhibition of ABC transporter Cdr1p by enniatin A.** DiS-C_3_(3) was added at the beginning of the experiment and fluorescence was measured every 4 min in strains with or without transporters; filled symbols indicate enniatin A addition (2 μg/ml) at 80 min, 2% glucose was added to the sample at 60 min (*N* = 3–4).

Fluorescent probes enabling the measurement of transporter activity in real time are valuable tools for screening new pump inhibitors. We tested the influence of a known inhibitor of Cdr1, enniatin A, on the transporter activity measured by fluorescence (Figures 2A,B). Addition of enniatin A to yeast cells resulted in a red shift of the fluorescence maximum of the probe in strains expressing Cdr1p, showing that the inhibitor is specific for Cdr1p and does not affect the activity of Cdr2p (Figure 2B).

**FIGURE 3:**
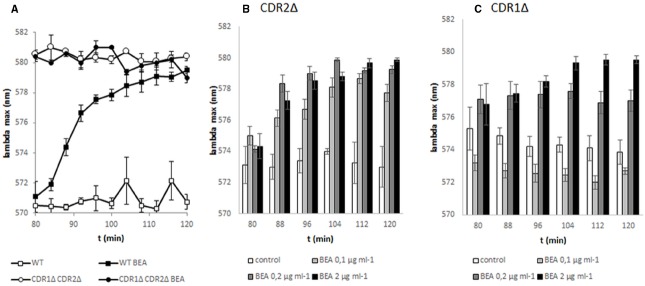
**Inhibition of ABC transporters Cdr1p and Cdr2p by beauvericin.** DiS-C_3_(3) was added at the beginning of the experiment and fluorescence was measured every 4 min. Two percent glucose was added to the sample at 60 min and beauvericin at 80 min. **(A)** Influence of 1 μg/ml beauvericin on diS-C_3_(3) staining in *C. albicans* WT and CDR1*Δ*CDR2*Δ* strains. **(B)** Dose-dependent inhibition of diS-C_3_(3) staining by beauvericin on *C. albicans* CDR1*Δ* strain. Lower lambda max indicates more probe transported out of the cells. **(C)** Dose-dependent inhibition of diS-C_3_(3) staining by beauvericin on *C. albicans* CDR2*Δ* strain.

After validation of the method with enniatin A, we tested the specificity of a new *C. albicans* CDR pump inhibitor, beauvericin (Figure 3). This inhibitor has been found to increase cell sensitivity to miconazole (Fukuda et al., 2004). But, to our knowledge, its specificity toward *C. albicans* transporters has never been tested.

In contrast to enniatin A, which affects the activity of only Cdr1p (Figure 2), beauvericin inhibited the activity of both Cdr1p and Cdr2p (Figure 3). As shown by diS-C_3_(3) efflux, Cdr1p was more sensitive to beauvericin than Cdr2p.

**FIGURE 4:**
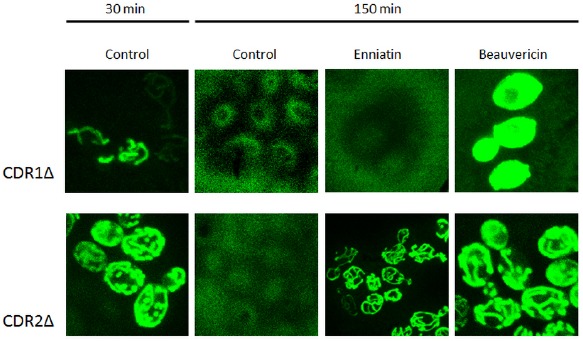
**Inhibition of diS-C_3_(3) export by enniatin A and beauvericin.** Staining was observed by confocal microscopy at 30 min (probe loaded into cells) and 150 min (full effect of the inhibitors).

To observe the activity of enniatin A and beauvericin as inhibitors of ABC transporters in real time, we monitored the accumulation of diS-C_3_(3) in *C. albicans* strains which express all pumps or lack Cdr1 and Cdr2 pumps using the confocal microscopy (Figure 4). We observed similar results to those obtained with the fluorimeter. The strain without Cdr1p pumped diS-C_3_(3) out the cell faster than the strain without Cdr2p. In strain without Cdr2p the fluorescence is visible after 30 min while in strain without Cdr1 diS-C_3_(3) is mainly present outside the cell (Figure 4). This confirms that Cdr2p plays a larger role in lowering of the probe concentration from the cells than Cdr1p.

After application of beauvericin the probe accumulated in both the *CDR1Δ* and *CDR2Δ* strains, unlike enniatin A in which activity as an inhibitor of probe efflux was observed only in the *CDR2Δ* strain (Figure 4). This result confirms our observation that beauvericin inhibited the activity of both Cdr1p and Cdr2p.

**FIGURE 5:**
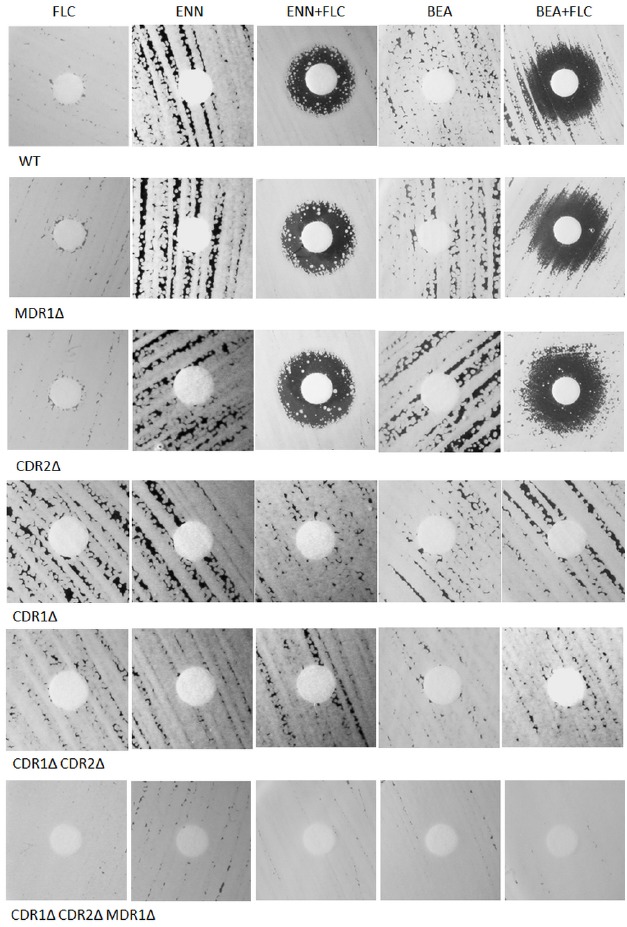
**Disk diffusion chemosensitization assays.** Paper disks containing inhibitors (2 μg of enniatin A; 10 μg beauvericin; 1.25 μg fluconazole for WT, MDR1*Δ*, and CDR2*Δ*; 0.15625 μg fluconazole for the three remaining strains) were placed on the surface of agarose plates seeded with yeast and the plates were incubated at 28 °C for 48 h.

Inhibition of *C. albicans* transporters observed by using the fluorescent probe diS-C_3_(3) should enable the screening for new drugs. We performed disk diffusion chemosensitization assays: paper disks containing fluconazole, alone, inhibitors (enniatin A, beauvericin), or combination of both were placed on plates seeded with *C. albicans* (Figure 5). The concentration of fluconazole was matched to the strain sensitivity so that it did not generate a growth inhibition zone. In case of strains expressing Cdr1p (*C. albicans* WT, *C. albicans MDR1Δ* or *C. albicans CDR2Δ*) inhibition zones were observed after addition of enniatin A or beauvericin together with fluconazole. This effect was not observed when the strains without Cdr1p were used. The combination of enniatin A with fluconazole increased the sensitivity of the strains in the same way shown by the fluorescence measurements (Figure 2). Beauvericin did inhibit probe export by both Cdr1 and Cdr2 (Figure 2), but it increased the sensitivity of the strain without Cdr2, not the strain without Cdr1 to fluconazole (Figure 5). This show further differences in the specificity of the inhibitors against diS-C_3_(3) and fluconazole.

## DISCUSSION

Multidrug resistance is a feature that causes serious medical problems associated with an increasing prevalence and diversity of fungal infections. This process was at first studied in the non-pathogenic yeast *S. cerevisiae* (Kolaczkowska and Goffeau, 1999; Rogers et al., 2001). But recently, investigators are focusing on pathogenic fungi such as *C. albicans*, *Candida glabrata*, or *Candida parapsilosis* (Morschhäuser, 2010).

The fluorescent probe diS-C_3_(3) has been found to be a useful tool to estimate and to continuously follow changes of the plasma membrane potential (PMP) of whole *S. cerevisiae* cells (Gášková et al., 1999), as well as to measure the kinetics of PDR pumps (Hendrych et al., 2009).

Our results indicate that diS-C_3_(3) may be useful in measuring the activity of PDR transporters in *C. albicans* as well. The diS-C_3_(3) probe is a substrate of Cdr1p and Cdr2p, but not Mdr1p. Previous investigations suggest that high aromatic, molecular branching compounds are substrates for Cdr1p, probably because of interactions with a large number of aromatic residues at an active site of the transporter (Puri et al., 2010). The possibility of observing the activity of efflux pumps in real time could provide a new tool for obtaining the answers to as yet unresolved-questions like the speed of changes in pump activity in response to environmental factors (e.g., substrates or inhibitors).

DiS-C_3_(3) easily passes through the plasma membrane and accumulates in the cells in response to membrane potential (Gášková et al., 1999). The staining of *C. albicans* strains by diS-C_3_(3) is approximately twice as slow as that of *S. cerevisiae* (Figures 1A,B; Hendrych et al., 2009). The reason for this difference in the rate of staining could be a lower PMP in *C. albicans* cells relative to *S. cerevisiae* cells. In the *S. cerevisiae* US 50–18C strain, with an overexpression of major pumps Pdr5p, Snq2p, and Yor1p, the cell ATP level varies depending on the growth phase and activity of PDR pumps (Krasowska et al., 2010). This is not the case in *C. albicans*.

The efflux of diS-C_3_(3) from *Candida* cells was inhibited by the depsipeptides, enniatin A, and beauvericin (Figures 2 and 3). Hiraga et al. (2005) suggested that enniatin A is a potent and specific inhibitor for Pdr5p, and Holmes et al. (2008) complemented these data by showing that enniatin A functions as an inhibitor of Cdr1p. Beauvericin was been found to function as an inhibitor of miconazole efflux from *C. albicans* (Fukuda et al., 2004). Enniatin A and beauvericin are ionophores that enhance the permeability of the cell membranes for ions (Tonshin et al., 2010). As shown here by the inhibition of diS-C_3_(3) efflux, both enniatin A and beauvericin interact with ABC transporters (Figure 3). But beauvericin, in contrast to enniatin A, shows a different synergism in case of fluconazole susceptibility (Figure 5). Similar differences in inhibitor activity were observed for curcumin (Sharma et al., 2009), the modulatory effect of which was restricted to rhodamine 6G or miconazole while it had no effect on the efflux of fluconazole. Indeed, most of inhibitors like enniatin A (Holmes et al., 2008), FK506 (Niimi et al., 2004), or curcumin (Sharma and Prasad, 2011) inhibit only Cdr1p. It seems that only tetrandrine blocks all Cdr1, Cdr2, and Mdr1 pumps (Zhang et al., 2009). To our knowledge, our report is the first to show that beauvericin is an inhibitor of not only Cdr1p but also of Cdr2p. The response of transporter activity was fast (visible after 12 min when examining gene transcription, as well as in cell staining) yet about 40 min were necessary to reach full activity.

## CONCLUSION

In this paper we show that the carbocyanine dye diS-C_3_(3) is employed in monitoring of real time activity of *C. albicans* ABC transporters Cdr1 and Cdr2. This method can be used as a powerful tools in the fight against multidrug resistance. Furthermore we present that two depsipeptides: enniatin A and beauvericin act as inhibitors of Cdr1p or Cdr1p and Cdr2p, respectively.

### Conflict of Interest Statement

The authors declare that the research was conducted in the absence of any commercial or financial relationships that could be construed as a potential conflict of interest.
